# Cost-Effectiveness of First-Line Versus Second-Line Pembrolizumab or Chemotherapy in Patients With Microsatellite-Instability-High/Mismatch Repair-Deficient Advanced Colorectal Cancer

**DOI:** 10.3389/fphar.2021.802942

**Published:** 2021-12-14

**Authors:** Tan Chongqing, Li Sini, Zeng Xiaohui, Peng Liubao, Peng Ye, Qin Shuxia, Wang Liting, Wu Meiyu, Wan Xiaomin

**Affiliations:** ^1^ Department of Pharmacy, The Second Xiangya Hospital, Central South University, Changsha, China; ^2^ Institute of Clinical Pharmacy, Central South University, Changsha, China; ^3^ School of Health and Related Research, Faculty of Medicine, Dentistry and Health, University of Sheffield, Sheffield, United Kingdom; ^4^ PET Imaging Center, The Second Xiangya Hospital, Central South University, Changsha, China

**Keywords:** pembrolizumab, colorectal neoplasms, immunotherapy, cost-effectiveness, microsatellite-instability-high/mismatch repair-deficient

## Abstract

**Background:** Pembrolizumab is a guideline-recommended, both first- and second-line treatment option for microsatellite-instability-high (MSI-H)/mismatch repair-deficient (dMMR)advanced colorectal cancer patients. The aim of the present study is to investigates the health and economic outcomes of three treatment strategies with or without pembrolizumab in MSI-H/dMMR advanced colorectal cancer to define the best treatment strategy from the perspective of the US payer.

**Methods:** A microsimulation model was developed to estimate the cost and effectiveness of three treatment strategies: 1) pembrolizumab used as first-line, 2) pembrolizumab used as second-line and, 3) chemotherapy. Life years (LYs), quality-adjusted LYs (QALYs) and lifetime costs were estimated.

**Results:** The model projected that patients receiving pembrolizumab in the first-line setting gained 5.579 QALYs; this value was 1.501 and 3.941 QALYs more than that for patients receiving pembrolizumab in the second-line setting and chemotherapy, respectively. First-line pembrolizumab strategy dominated second-line pembrolizumab strategy. Compared with chemotherapy, first-line pembrolizumab strategy yielded an incremental cost of $50613.7, which resulted in an ICER of $13441 per QALY.

**Conclusion:** For patients with MSI-H/dMMR advanced colorectal cancer, reserving pembrolizumab for second-line line use is dominated by its first-line use, and first-line use of pembrolizumab is cost-effective compared with chemotherapy.

## Introduction

Colorectal cancer is the third most common cancer and the second leading cause of cancer death in the United State (Siegel et al., 2020). Microsatellite-instability-high (MSI-H)/mismatch repair-deficient (dMMR) represents a subtype that occurs in about 4% of patients with advanced disease (Casak et al., 2021). MSI-H/dMMR tumors are less responsive to chemotherapy, however, chemotherapy remains the standard of care for patients with MSI-H/dMMR advanced colorectal cancer (Innocenti et al., 2019).

The advent of immune checkpoint inhibitors has altered the therapeutic landscape of MSI-H/dMMR advanced colorectal cancer. In 2017, programmed death 1 (PD-1) inhibitors pembrolizumab received the first tumor-type-agnostic approval from the US Food and Drug Administration (FDA)for the treatment of patients with MSI-H/dMMR solid tumors that has progressed following prior chemotherapy (Marcus et al., 2019). More recently, the landmark KEYNOTE-177 phase 3 trial, investigating pembrolizumab as the first-line therapy for MSI-H/dMMR advanced colorectal cancer, has shown improved outcomes, with a median progression-free survival more than twice as long in patients receiving pembrolizumab compared with chemotherapy (16.5 vs. 8.2 months, respectively) (André et al., 2020). Based on these results, pembrolizumab was approved by the FDA and is a guideline-recommended, first-line treatment option for MSI-H/dMMR advanced colorectal cancer patients and represents the new standard-of-care (Casak et al., 2021; National Comprehensive Cancer Network, 2021).

Although pembrolizumab used in the first-line setting significantly prolonged progression-free survival compared with chemotherapy, pembrolizumab is expensive, with cost estimates of $350,000 for patients who completed 35 treatments (CMS, 2021a). Whether or not the use of pembrolizumab would be cost-effective is unclear.

The aim of the present study is to investigates the health and economic outcomes of three treatment strategies with or without pembrolizumab in MSI-H/dMMR advanced colorectal cancer to define the best treatment strategy from the perspective of the US payer.

## Methods

### Model Overview

**FIGURE 1:**
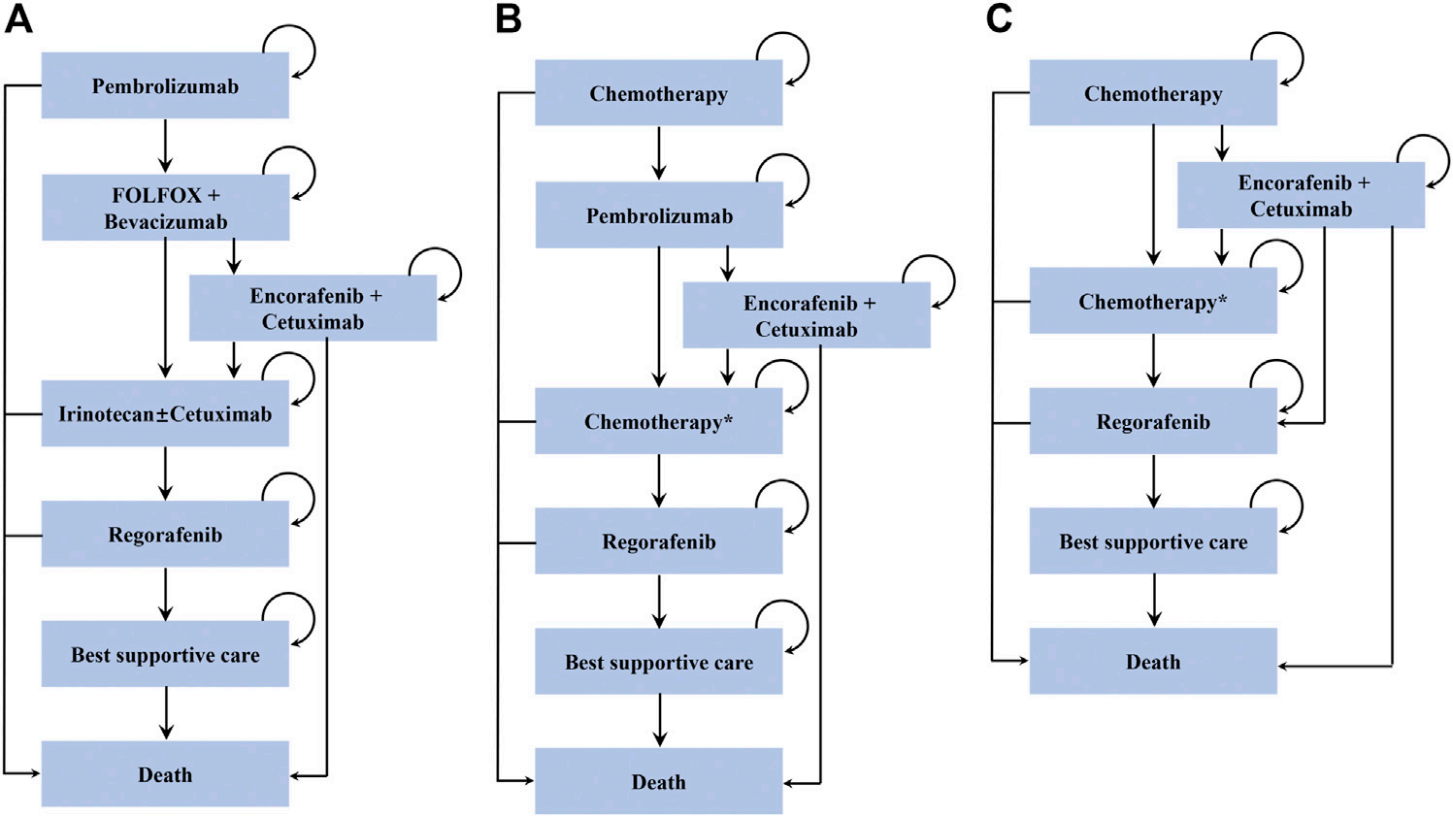
Treatment sequences used in the model. **(A)** Treatment sequence for patients who receive pembrolizumab in the first-line setting. **(B)** Treatment sequence for patients who receive pembrolizumab in the second-line setting. **(C)** Treatment sequence for patients who receive chemotherapy.

We used a microsimulation model to analyze the cost-effectiveness of MSI-H/dMMR advanced colorectal cancer (Supplementary Figure S1). A hypothetical cohort of 10,000 patients was modeled with baseline characteristics similar to the cohort enrolled in the KEYNOTE-177 trial (Table 1) (André et al., 2020). A total of three treatment strategies were evaluated in the model (Figure 1): 1) pembrolizumab used as first-line, 2) pembrolizumab used as second-line and, 3) chemotherapy. Individual patients entered the model with newly diagnosed MSI-H/dMMR advanced colorectal cancer and received either first-line pembrolizumab or physician’s choice chemotherapy, including FOLFOX or FOLFIRI with or without bevacizumab or cetuximab (Table 1) (André et al., 2020). The subsequent treatment sequence was assumed according to The National Comprehensive Cancer Network (NCCN) Clinical Practice Guidelines (National Comprehensive Cancer Network, 2021). In pembrolizumab-containing treatment arms, patients who progressed on first-line pembrolizumab subsequently received FOLFOX plus bevacizumab (Giantonio et al., 2007), while those progressing on first-line chemotherapy subsequently received pembrolizumab (Le et al., 2020). After progression, patients with BRAF V600E mutant received encorafenib plus cetuximab (Tabernero et al., 2021). Patients without BRAF V600E mutant or patients whose cancer progressed on encorafenib plus cetuximab in the first-line pembrolizumab arm subsequently irinotecan with or without cetuximab (Sobrero et al., 2008) and regorafenib (Grothey et al., 2013), while those without BRAF V600E mutant or those progressing on encorafenib plus cetuximab in the second-line pembrolizumab arm subsequently received chemotherapy (Giantonio et al., 2007; Tabernero et al., 2015) and regorafenib (Grothey et al., 2013). The choice of the chemotherapy on progression after second-line pembrolizumab depended on the first-line chemotherapy (ie, chemotherapy in patients who were given first-line FOLFIRI was switched with third-line FOLFOX plus bevacizumab and vice versa). In chemotherapy arm, treatment therapies were the same as those in second-line pembrolizumab arm with the exception that patients who received FOLFOX as first-line treatment and progressed on encorafenib plus cetuximab subsequently received regorafenib in chemotherapy arm (Grothey et al., 2013). Patients who experienced progression after regorafenib received a best supportive care before death.

**TABLE 1:** Model clinical parameters.

Parameter	Estimate	Range	Distribution	Reference
PFS for pembrolizumab first-line therapy	Spline (knot = 3): gamma0 = −3.340736, gamma1 = 0.955158, gamma2 = −5.802732, gamma3 = 6.926259, gamma4 = −1.171400	—	Multivariable normal	André et al. (2020)
PFS for encorafenib plus cetuximab	Loglogistic: shape = 2.116512, scale = 4.660681	—	Multivariable normal	Tabernero et al. (2021)
PFS for FOLFOX plus bevacizumab	Weibull: a = 1.576, b = 9.101	—	Multivariable normal	Giantonio et al. (2007)
PFS for irinotecan	Spline (knot = 2): gamma0 = −2.2013510, gamma1 = 3.4960096, gamma2 = 0.5627302, gamma3 = −0.3947207	—	Multivariable normal	Sobrero et al. (2008)
PFS for cetuximab plus irinotecan	Spline (knot = 3): gamma0 = −1.7244692, gamma1 = 4.5958398, gamma2 = 1.0911,644, gamma3 = −1.4293002, gamma4 = 0.6907344		Multivariable normal	Sobrero et al. (2008)
PFS for chemotherapy first-line therapy	Generalized gamma: mu = 1.81749713, sigma = 0.05360466, Q = −0.61570022	—	Multivariable normal	André et al. (2020)
PFS for pembrolizumab second-line therapy	Spline (knot = 3): gamma0 = −3.4041439, gamma1 = 0.6674260, gamma2 = −4.1798710, gamma3 = 5.1161971, gamma4 = −0.9710231	—	Multivariable normal	Le et al. (2020)
PFS for FOLFIRI	Spline (knot = 3): gamma0 = −1.1819358, gamma1 = 4.7007055, gamma2 = 0.7169704, gamma3 = -1.2818196, gamma4 = 0.7828784	—	Multivariable normal	Tabernero et al. (2015)
PFS for FOLFOX	Generalized gamma: mu = 1.6748, sigma = 0.7089, Q = 0.3498	—	Multivariable normal	Giantonio et al. (2007)
PFS for regorafenib	Spline (knot = 3): gamma0 = −4.6041562, gamma1 = 0.6411741, gamma2 = −11.4902184, gamma3 = 13.9327194, gamma4 = −2.7779302	—	Multivariable normal	Grothey et al. (2013)
Median survival time in best supportive care, days	28.5	14–42	Exponential: lambda = 0.51074	Connor et al. (2007), Christakis and Escarce (1996)
Proportion of patients in first-line chemotherapy arm who received FOLFOX or FOLFIRI with or without bevacizumab or cetuximab				
FOLFOX	0.08	—	—	André et al. (2020)
FOLFOX plus bevacizumab	0.45	—	—	André et al. (2020)
FOLFOX plus cetuximab	0.03	—		André et al. (2020)
FOLFIRI	0.11	—	—	André et al. (2020)
FOLFIRI + bevacizumab	0.25	—	—	André et al. (2020)
FOLFIRI + cetuximab	0.08	—	—	André et al. (2020)
Probability of discontinuing treatment due to AE, %				
Pembrolizumab first-line therapy	14.40	7.20–21.60	Beta: a = 22, b = 131	André et al. (2020)
Encorafenib plus cetuximab	9.00	4.50–13.50	Beta: a = 9,b = 91	Tabernero et al. (2015)
FOLFOX plus bevacizumab	23.40	11.70–35.10	Beta: a = 23,b = 77	Giantonio et al. (2007)
Irinotecan	4.77	2.39–7.16	Beta: a = 31,b = 619	Sobrero et al. (2008)
Cetuximab plus irinotecan	6.50	3.25–9.75	Beta: a = 42,b = 606	Sobrero et al. (2008)
Chemotherapy first-line therapy	11.90	5.95–17.85	Beta: a = 17,b = 126	André et al. (2020)
Pembrolizumab second-line therapy	6.35	3.18–9.53	Beta: a = 4,b = 59	Le et al. (2020)
FOLFIRI	4.29	2.15–6.44	Beta: a = 23,b = 513	Tabernero et al. (2015)
FOLFOX	23.90	11.95–35.85	Beta: a = 24,b = 76	Giantonio et al. (2007)
Regorafenib	8.40	4.20–12.60	Beta: a = 42,b = 458	Grothey et al. (2013)
Probability of mortality due to AE, %				
Pembrolizumab first-line therapy	0	—	—	André et al. (2020)
Encorafenib plus cetuximab	4.00	2–6	Beta: a = 4,b = 96	Tabernero et al. (2021)
FOLFOX plus bevacizumab	5.00	2.5–7.5	Beta: a = 5,b = 95	Giantonio et al. (2007)
Irinotecan	0.31	0.16–0.47	Beta: a = 2,b = 648	Sobrero et al. (2008)
Cetuximab plus irinotecan	0.77	0.39–1.16	Beta: a = 5,b = 645	Sobrero et al. (2008)
Chemotherapy first-line therapy	0.70	0.35–1.05	Beta: a = 1,b = 142	André et al. (2020)
Pembrolizumab second-line therapy	0	—	—	Le et al. (2020)
FOLFIRI	2.05	1.03–3.08	Beta: a = 11,b = 525	Tabernero et al. (2015)
FOLFOX	4.00	2–6	Beta: a = 4,b = 96	Giantonio et al. (2007)
Regorafenib	1.60	0.8–2.4	Beta: a = 8,b = 492	Grothey et al. (2013)
Incidence of Grade 1 or 2 AE, %				
pembrolizumab first-line therapy	0.41	0.21–0.62	Beta: a = 41,b = 59	André et al. (2020)
Encorafenib plus cetuximab	0.41	0.21–0.62	Beta: a = 40,b = 60	Tabernero et al. (2021)
FOLFOX plus bevacizumab	0.25	0.13–0.38	Beta: a = 25,b = 75	Giantonio et al. (2007)
Irinotecan	0.53	0.27–0.80	Beta: a = 52,b = 48	Sobrero et al. (2008)
Cetuximab plus irinotecan				Sobrero et al. (2008)
Chemotherapy first-line therapy	0.21	0.11–0.32	Beta: a = 21,b = 79	André et al. (2020)
Pembrolizumab second-line therapy	0.57	0.29–0.86	Beta: a = 57,b = 43	Le et al. (2020)
FOLFIRI	0.27	0.14–0.41	Beta: a = 27,b = 73	Tabernero et al. (2015)
FOLFOX	0.39	0.20–0.59	Beta: a = 39,b = 61	Giantonio et al. (2007)
Regorafenib	0.39	0.20–0.59	Beta: a = 39,b = 61	Grothey et al. (2013)
Incidence of Grade 3 or greater AE, %				
Pembrolizumab first-line therapy	0.56	0.28–0.84	Beta: a = 56,b = 44	André et al. (2020)
Encorafenib plus cetuximab	0.58	0.29–0.87	Beta: a = 57,b = 43	Tabernero et al. (2021)
FOLFOX plus bevacizumab	0.75	0.38–1	Beta: a = 75,b = 25	Giantonio et al. (2007)
Irinotecan	0.44	0.22–0.66	Beta: a = 43,b = 57	Sobrero et al. (2008)
Chemotherapy first-line therapy	0.78	0.39–1.17	Beta: a = 78,b = 22	Sobrero et al. (2008)
Pembrolizumab second-line therapy	0.13	0.07–0.20	Beta: a = 13,b = 87	André et al. (2020)
FOLFIRI	0.72	0.36–1.08	Beta: a = 72,b = 28	Le et al. (2020)
FOLFOX	0.61	0.31–0.92	Beta: a = 61,b = 39	Tabernero et al. (2015)
Regorafenib	0.54	0.27–0.81	Beta: a = 54,b = 46	Giantonio et al. (2007)
Patient characteristics at baseline				Grothey et al. (2013)
Age	63	24–93	Truncated Normal: mean = 63, sd = 17.6, lower = 24, upper = 93	André et al. (2020)
Male sex, %	46	—	—	André et al. (2020)
Wight, kg				
Male	90	—	—	CDC (2021)
Female	77	—	—	CDC (2021)
Body surface area, m^2^				
Male	1.9	—	—	CDC (2021)
Female	1.6	—	—	CDC (2021)
BRAF^V600E^ mutant, %	0.22	0.11–0.33	Uniform (0.11, 0.33)	André et al. (2020)

Each model cycle represented 3 weeks. A lifetime horizon was used to estimate the costs and effectiveness associated with each treatment strategy. A 3% discount rate per year was adopted for both costs and effectiveness. The outputs included the total cost, life-years (LYs), quality-adjusted LYs (QALYs), and incremental cost effectiveness ratios (ICERs). A willingness-to-pay (WTP) threshold of$150,000 per QALY as recommended by Neumann et al. was used to determine the cost-effectiveness of therapy (Neumann et al., 2014).

The development of the microsimulation model was performed using R statistical software (version 4.0.2; http://www.r-proje ct.org). The model was validated following the ISPOR Task Force recommendation (Eddy et al., 2012).

### Clinical Data Inputs

The progression risks for each line of therapy were informed by the respective trial and extrapolated over the model time horizon. The GetData Graph Digitizer software package (version 2.25; http://www.getda ta-graph-digit izer. com/index.php) was used to extract progression free survival (PFS) probabilities from each PFS Kaplan-Meier curves of each trial. The algorithm derived by Guyot et al. (2012) was used to generate a pseudo-individual patient data, and parametric survival functions including exponential, Weibull, log-normal, log-logistic, generalized gamma, Gompertz, Royston/Parmar spline model, and parametric mixture cure models then were used to fit the pseudo-individual patient data. The final parametric survival function selected for inclusion in the model was based on the goodness of fit measured using the Akaike information criterion (Supplementary Table S1).

Treatment discontinuation due to adverse events (AEs) was also incorporated in the model, with the rates derived from the respective trial (Giantonio et al., 2007; Sobrero et al., 2008; Grothey et al., 2013; Tabernero et al., 2015; André et al., 2020; Le et al., 2020; Tabernero et al., 2021). Given the greater frequency of AEs in the first 2 months of treatment (Patel et al., 2021a), the treatment discontinuation was assumed to occur within the first 2 months of each line of therapy. The background mortality rate was derived from US life tables (Arias et al., 2019). Mortality rate for patients receiving the best supportive care was derived from a retrospective study of the Medicare database (Christakis and Escarce, 1996; Connor et al., 2007). Estimates for transition probabilities are listed in Table 1.

### Cost and Utility Estimates

All information regarding costs and utilities is listed in Table 2. The model included only direct medical care costs of drug, administration, and management of adverse events (AEs). The 2021 average sale price from the Centers for Medicare and Medicaid Services was used to estimate the costs of intravenous medications (CMS, 2021a). We used gender-specific weight and body surface area to calculate medication costs based on mean US values (Table 1) (CDC, 2021). Administration costs were estimated according to the 2021 Centers for Medicare and Medicaid Service Physician Fee Schedule (CMS, 2021b). The cost of oral medications, including encorafenib and regorafenib, were derived from a previously published study (Patel et al., 2021b). We considered the impact of both grade 1 or 2 and grade 3 or greater AEs in the model as measured by health disutility weight and AEs costs. A previous cost-effectiveness study of advanced colorectal cancer was used to estimate the costs of adverse events and best supportive care (Chu et al., 2019). Where appropriate, we adjusted costs for inflation to reflect 2021 US dollars using the US Consumer Price Index.

**TABLE 2:** Model costs and utilities.

Parameter	Estimate, $	Range	Distribution	Reference
Drug acquisition cost per cycle, $				
Pembrolizumab	10,129.6	—	—	CMS (2021a)
Encorafenib plus cetuximab				
First cycle	15,064.412	—	—	CMS (2021a), Patel et al. (2021b)
Subsequent cycle	13,492.82	—	—	CMS (2021a), Patel et al. (2021b)
FOLFOX plus bevacizumab	4,462.3	—	—	CMS (2021a)
Irinotecan	68.6	—	—	CMS (2021a)
Cetuximab plus irinotecan				
First cycle	9,498.124			CMS (2021a)
Subsequent cycle	7,926.532			CMS (2021a)
FOLFOX	122.0	—	—	CMS (2021a)
FOLFOX plus cetuximab				
First cycle	9,551.6	—	—	CMS (2021a)
Subsequent cycle	7,980.0	—	—	CMS (2021a)
FOLFIRI	128.0	—	—	CMS (2021a)
FOLFIRI plus bevacizumab	4,468.3	—	—	CMS (2021a)
FOLFIRI plus cetuximab				
First cycle	9,557.5	—	—	CMS (2021a)
Subsequent cycle	7,985.9	—	—	CMS (2021a)
Regorafenib	13,675.5	—	—	Patel et al. (2021b)
Cost of best supportive care, $/week	90	45–135	Gamma: shape = 3.84, scale = 0.014	Chu et al. (2019)
Administration cost, US$				
Chemotherapy IV infusion, first hour	148.3	—	—	CPT 96413
Chemotherapy IV infusion, additional hour	31.4	—	—	CPT 96415
Chemotherapy IV infusion additional sequence	71.9	—	—	CPT 96417
Chemotherapy IV push initial	113.4	—	—	CPT 96409
Chemotherapy prolong infuse w/pump	147.3	—	—	CPT 96416
AE cost, $ per event pembrolizumab				
Grade 1 or 2 AE	120.14	60.07–180.21	Gamma: shape = 1.90, scale = 63.29	Chu et al. (2019)
Grade 3 or greater AE	691.15	345.58–1,036.73	Gamma: shape = 0.73, scale = 227.60	Chu et al. (2019)
Other therapy				
Grade 1 or 2 AE	165.19	82.60–247.79	Gamma: shape = 7.67, scale = 90.07	Chu et al. (2019)
Grade 3 or greater AE	369.17	184.59–553.76	Gamma: shape = 0.36, scale = 1,015.22	Chu et al. (2019)
Utility				
First-line therapy				
pembrolizumab	0.84	0.67–1	Beta: a = 42,b = 8	Andre et al. (2021)
Chemotherapy	0.77	0.62–0.92	Beta: a = 38,b = 12	Andre et al. (2021)
Subsequent active therapies	0.65	0.52–0.78	Beta: a = 32,b = 18	Chu et al. (2019), Goldstein et al. (2015)
Best supportive care	0.35	0.28–0.42	Beta: a = 17,b = 33	Chu et al. (2019), Goldstein et al. (2015)
Grade 1 or 2 AE				
pembrolizumab in the second-line setting	0.09	0.07–0.10	Beta: a = 4,b = 46	Chu et al. (2019)
Other therapy	0.11	0.09–0.13	Beta: a = 5,b = 45	Chu et al. (2019)
Grade 3 or greater AE				
pembrolizumab in the second-line setting	0.17	0.13–0.20	Beta: a = 8,b = 42	Chu et al. (2019)
Other therapy	0.25	0.2–0.3	Beta: a = 12,b = 38	Chu et al. (2019)

QALYs in the model were calculated by adjusting survival time by health-related quality of life. We assigned a utility of 0.84 for patients receiving pembrolizumab in the first setting and 0.77 for those receiving chemotherapy in the first setting, respectively, based on quality-of-life data collected in the KEYNOTE-177 trial (Andre et al., 2021). A utility value of 0.65 and 0.35 from previously published economic evaluation was assigned for patients receiving subsequent active treatments and best supportive care, respectively (Goldstein et al., 2015; Chu et al., 2019). The loss in QALYs due to AEs was estimated by multiplying the incidences and corresponding disutility values of the AEs (Chu et al., 2019). Because the experiences of patients receiving first-line treatment were reflected in the utilities and, therefore, treatment-related AEs were included in the utilities, additional utility decrements associated with AEs were not modeled for first-line setting.

### Sensitivity Analysis

A series of sensitivity analyses were performed to explore the model uncertainty. The one-way sensitivity analysis was carried out by varying each parameter singly across the ranges listed in Tables 1, 2. In probabilistic sensitivity analysis, we performed 250 Monte Carlo samples of 5,000 patients with the parameters simultaneously varied with a specific pattern of distribution (Tables 1, 2). Based on the data from probabilistic sensitivity analysis, a cost-effectiveness acceptability curve was created to represent the probability that each treatment strategy is the most cost effective at various WTP thresholds. A scenario analysis was also performed in which we assumed that 10–30% of patients elected to receive best supportive care after progressing from second-line treatment.

## Results

### Model Validation

Simulated clinical outcomes in our model were consistent with the results of respective clinical trials in terms of PFS (Supplementary Figures S2–S6). Although the KEYNOTE-177 trial did not report OS, the median OS time of 1.84 years (undiscounted) for patients in chemotherapy arm, estimated by our model, matched the median OS time of 1.97 years reported in a systematic review which aggregated25 published trials on the efficacy of oxaliplatin-based plus bevacizumab as first-line treatment for advanced colorectal cancer (Petrelli et al., 2015).

### Base Case Results

The model projected that the life expectancy of patients receiving pembrolizumab as first-line therapy was 6.914 LYs, which was associated with an improvement of 0.823 and 4.557 LYs compared with second-line pembrolizumab and chemotherapy, respectively (Table 3). The average duration of pembrolizumab exposure was longer when used in the first-line setting compared with the second-line setting (10.5 vs. 9.6 months). Accounting for quality of life, patients receiving pembrolizumab in the first-line setting gained 5.579 QALYs; this value was 1.501 and 3.941 QALYs more than that for patients receiving pembrolizumab in the second-line setting and chemotherapy, respectively. First-line pembrolizumab strategy dominated second-line pembrolizumab strategy due to its lower cost and greater effectiveness. Compared with chemotherapy, first-line pembrolizumab strategy yielded an additional 3.941 QALYs with an incremental cost of $50613.7, which resulted in an ICER of $13441 per QALY.

**TABLE 3:** Base-case results.

Strategy	Cost, $	Effectiveness, LY	Effectiveness, QALY	ICER, $/QALY^a^
No pembrolizumab	186760.7	2.357	1.638	—
First-line pembrolizumab	237373.7	6.914	5.579	13,441
Second-line pembrolizumab	297500.7	6.091	4.078	Dominated

a The ICER, was compared with the next most effective non-dominated alternative.

Abbreviations; LY, life year; QALY, quality-adjusted life year; ICER, incremental cost-effectiveness ratio

### Sensitivity Analysis

**FIGURE 2:**
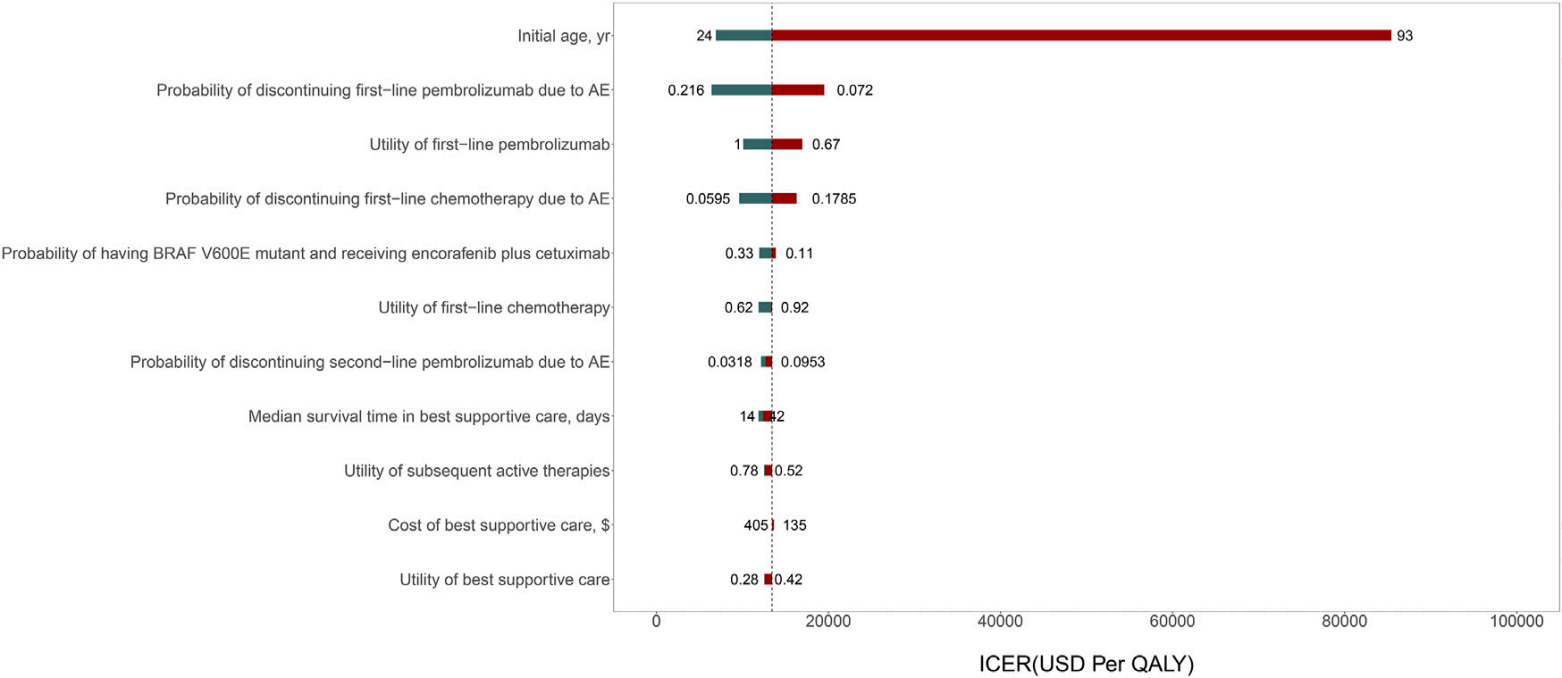
Tornado diagram of one-way sensitivity analysis of the incremental cost-effectiveness ratio (ICER) for first-line pembrolizumab versus chemotherapy.

**FIGURE 3:**
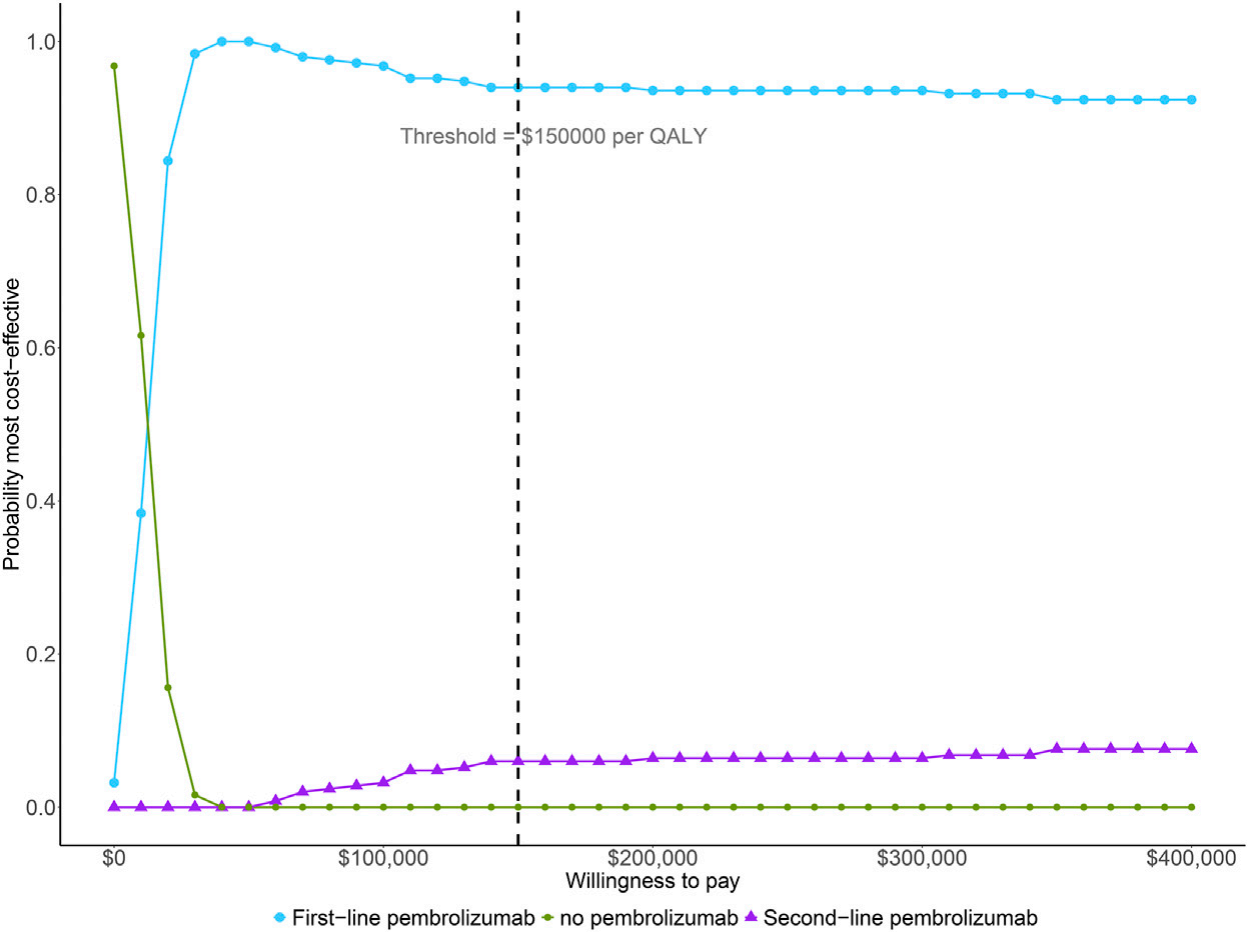
The cost-effectiveness acceptability curve simultaneously comparing the cost-effectiveness of three competing strategies. QALY, quality-adjusted life year.

The result of one-way sensitivity analyses of first-line pembrolizumab in comparison with chemotherapy is presented in Figure 2. The variables with greatest influence on the ICER was the patient’s initial age. Decreasing the initial age from 63 to 24 decreased the ICER to $6908 per QALY. On the other hand, increasing the initial age from 63 to 93 years increased the ICER to $85426 per QALY. Other variables had a moderate or minor influence on the ICER. Across the broad variations in the ranges for each parameter, the ICER for first-line pembrolizumab compared with chemotherapy remained <$100000 per QALY. The results of probabilistic sensitivity analyses suggested that, at a WTP threshold of $150000 per QALY, the probability of first-line pembrolizumab being cost-effective is greater than 90% in the simultaneous competition of the three treatment strategies (Figure 3).

Assuming that 10–30% of patients elected to receive best supportive care after progressing from second-line treatment had a minor influence on the results (Supplementary Table S2).

## Discussion

To our knowledge, this is the first cost-effectiveness analysis of pembrolizumab for patients with MSI-H/dMMR advanced colorectal cancer. Our model suggested that first-line use of pembrolizumab is cost-effective compared with chemotherapy and second-line use of pembrolizumab was dominated because of its higher cost and lower health outcomes. The probabilistic sensitivity analysis revealed that the probability of first-line use of pembrolizumab being cost-effective was greater than 90% for almost all reasonable WTP thresholds.

Chu et al. have analyzed the cost-effective of nivolumab with and without ipilimumab in the third-line setting for patients with MSI-H/dMMR advanced colorectal cancer from a US third-party payer perspective (Chu et al., 2019). They found that neither nivolumab alone nor nivolumab plus ipilimumab was cost-effective compared with trifluridine and tipiracil. This is driven by its infinite use of nivolumab until disease progression or unacceptable toxicity (Chu et al., 2019). Restricting the duration of nivolumab treatment to 2 years resulted in an ICER well under a threshold of $100000 per QALY (Chu et al., 2019). Given the finite course of pembrolizumab, it is not surprising that our findings with regard to the cost-effectiveness analysis of pembrolizumab were not in keeping with those by Chu et al. This may be a potential reason for pembrolizumab being a preferred treatment option recommended by the National Comprehensive Cancer Network for Colon Cancer (National Comprehensive Cancer Network, 2021).

This study has several strengths worth highlighting. First, the majority of clinical data inputs were derived from large and well-designed randomized phase 3 clinical trials (Giantonio et al., 2007; Sobrero et al., 2008; Grothey et al., 2013; Tabernero et al., 2015; André et al., 2020; Le et al., 2020; Tabernero et al., 2021). Second, the use of encorafenib plus cetuximab for patients with BRAF V600E-Mutant was incorporated in the model (Tabernero et al., 2021). Finally, we accounted for treatment discontinuation and mortality due to AEs in the model as well as AE-related costs and disutility.

As with any model, there are also limitations to our analysis. First, it was assumed that patients received specific sequences of therapies after first-line treatment based on NCCN guidelines. In practice, the choice of subsequent therapy may be affected by several factors, such as patient situations, and can differ from our assumption. The results of scenario analysis, however, demonstrated that the changes of subsequent therapy minimally changed the model. Second, the efficacy of pembrolizumab in second-line setting was derived from a nonrandomized, phase II, single arm trial (KEYNOTE-164) (Le et al., 2020). Our model, consequently, is essentially reliant on the validity of the trial and any biases within the trial will be reflected in the model. Third, owing to the lack of the survival data of patients with MSI-H/dMMR advanced colorectal cancer who received second- or further-line chemotherapy (André et al., 2020), clinical data used to populate the model was derived from trials which contained only a small percentage of patients with MSI-H/dMMR or patients with microsatellite instability/mismatch repair status unknown. AsMSI-H–dMMR tumors are less responsive to chemotherapy (André et al., 2020), this may have resulted in overestimation of the survival outcome in our model. However, given that second- or further-line chemotherapy outcomes were similar across each treatment group, this limitation is not expected to substantially influence the incremental effectiveness or the ICER. Finally, lifetime outcomes and costs were estimated based on multiple clinical trials which slightly differed inpatient population.

## Conclusion

From the perspective of the US payer, for patients with MSI-H/dMMR advanced colorectal cancer, our study suggests that reserving pembrolizumab for second-line line use is dominated by its first-line use, and first-line use of pembrolizumab is cost-effective compared with chemotherapy at a WTP threshold of $150,000 per QALY.

## Data Availability

The original contributions presented in the study are included in the article/Supplementary Material, further inquiries can be directed to the corresponding author.
