# Supplementary material for: Cost-Effectiveness of First-Line Versus Second-Line Pembrolizumab or Chemotherapy in Patients With Microsatellite-Instability-High/Mismatch Repair-Deficient Advanced Colorectal Cancer
Source: Front Pharmacol. 2021 Dec 14;12:802942. doi: 10.3389/fphar.2021.802942 (PMC8712714; doi:10.3389/fphar.2021.802942)

Data Supplement for “Cost-effectiveness of pembrolizumab in patients with microsatellite-instability-high/mismatch repair-deficient advanced colorectal cancer”

eTable 1 The results of goodness-of-fit

| Trial | Treatment regimens | Distribution | AIC |
| --- | --- | --- | --- |
| KEYNOTE-177 | pembrolizumab in first-line setting | exp | 586.8579 |
|  |  | weibull | 561.8935 |
|  |  | lognorm | 544.3135 |
|  |  | llogis | 551.1364 |
|  |  | gompertz | 533.1490 |
|  |  | ggamma | 531.8440 |
|  |  | spline with knot=1 | 520.0540 |
|  |  | spline with knot=2 | 521.2214 |
|  |  | spline with knot=3 | 505.9117 |
|  |  | MCM with exp | 534.8099 |
|  |  | MCM with weibull | 536.8064 |
|  |  | MCM with ggamma | 525.7623 |
|  |  | MCM with llogis | 520.7091 |
|  |  | MCM with gompertz | 533.0683 |
|  |  | MCM with lognorm | 523.8644 |
|  | chemotherapy first-line therapy | exp | 800.4761 |
|  |  | weibull | 801.5115 |
|  |  | lognorm | 782.9968 |
|  |  | llogis | 788.6162 |
|  |  | gompertz | 801.8292 |
|  |  | ggamma | 781.7114 |
|  |  | spline with knot=1 | 785.6722 |
|  |  | spline with knot=2 | 782.8258 |
|  |  | spline with knot=3 | 784.2932 |
|  |  | MCM with exp | 801.6272 |
|  |  | MCM with weibull | 799.4245 |
|  |  | MCM with ggamma | 783.7155 |
|  |  | MCM with llogis | 790.4802 |
|  |  | MCM with gompertz | 803.6080 |
|  |  | MCM with lognorm | 784.7861 |
| BEACON | encorafenib plus cetuximab | exp | 927.6079 |
|  |  | weibull | 901.2204 |
|  |  | lognorm | 883.9545 |
|  |  | llogis | 883.7300 |
|  |  | gompertz | 922.9426 |
|  |  | ggamma | 885.7246 |
|  |  | spline with knot=1 | 885.7279 |
|  |  | spline with knot=2 | 887.7845 |
|  |  | spline with knot=3 | 889.4992 |
|  |  | MCM with exp | 929.6119 |
|  |  | MCM with weibull | 890.0562 |
|  |  | MCM with ggamma | 885.9599 |
|  |  | MCM with llogis | 885.5714 |
|  |  | MCM with gompertz | 908.2260 |
|  |  | MCM with lognorm | 885.6157 |
| E3200 | FOLFOX plus bevacizumab | exp | -559.2304 |
|  |  | weibull | -1189.1591 |
|  |  | lognorm | -963.2056 |
|  |  | llogis | -973.3665 |
|  |  | gompertz | -1012.3265 |
|  |  | ggamma | -1188.0509 |
|  | FOLFOX | exp | -1039.862 |
|  |  | weibull | -2099.688 |
|  |  | lognorm | -2289.426 |
|  |  | llogis | -2262.643 |
|  |  | gompertz | -1682.014 |
|  |  | ggamma | -2397.394 |
| EPIC | irinotecan | exp | 2149.878 |
|  |  | weibull | 2091.270 |
|  |  | lognorm | 2052.389 |
|  |  | llogis | 2064.702 |
|  |  | gompertz | 2137.376 |
|  |  | ggamma | 2053.572 |
|  |  | spline with knot=1 | 2056.565 |
|  |  | spline with knot=2 | 2040.236 |
|  |  | spline with knot=3 | 2045.115 |
|  |  | MCM with exp | 2151.885 |
|  |  | MCM with weibull | 2084.215 |
|  |  | MCM with ggamma | 2055.584 |
|  |  | MCM with llogis | 2066.777 |
|  |  | MCM with gompertz | 2128.172 |
|  |  | MCM with lognorm | 2054.423 |
|  | cetuximab plus irinotecan | exp | 3098.206 |
|  |  | weibull | 3049.095 |
|  |  | lognorm | 2996.873 |
|  |  | llogis | 3015.281 |
|  |  | gompertz | 3094.226 |
|  |  | ggamma | 2996.777 |
|  |  | spline with knot=1 | 3004.928 |
|  |  | spline with knot=2 | 2990.304 |
|  |  | spline with knot=3 | 2972.085 |
|  |  | MCM with exp | 3097.969 |
|  |  | MCM with weibull | 3025.429 |
|  |  | MCM with ggamma | 2997.165 |
|  |  | MCM with llogis | 3017.318 |
|  |  | MCM with gompertz | 3071.232 |
|  |  | MCM with lognorm | 2998.862 |
| KEYNOTE-164 | pembrolizumab second-line therapy | exp | 294.7872 |
|  |  | weibull | 278.8204 |
|  |  | lognorm | 269.1438 |
|  |  | llogis | 270.9753 |
|  |  | gompertz | 258.9550 |
|  |  | ggamma | 266.8190 |
|  |  | spline with knot=1 | 256.4624 |
|  |  | spline with knot=2 | 252.8852 |
|  |  | spline with knot=3 | 247.6237 |
|  |  | MCM with exp | 259.0706 |
|  |  | MCM with weibull | 260.9377 |
|  |  | MCM with ggamma | 259.7992 |
|  |  | MCM with llogis | 254.5886 |
|  |  | MCM with gompertz | 260.1121 |
|  |  | MCM with lognorm | 259.1464 |
| RAISE | FOLFIRI | exp | 2721.639 |
|  |  | weibull | 2685.156 |
|  |  | lognorm | 2647.437 |
|  |  | llogis | 2648.530 |
|  |  | gompertz | 2718.317 |
|  |  | ggamma | 2645.507 |
|  |  | spline with knot=1 | 2645.565 |
|  |  | spline with knot=2 | 2646.470 |
|  |  | spline with knot=3 | 2635.146 |
|  |  | MCM with exp | 2723.645 |
|  |  | MCM with weibull | 2675.384 |
|  |  | MCM with ggamma | 2647.412 |
|  |  | MCM with llogis | 2650.585 |
|  |  | MCM with gompertz | 2713.778 |
|  |  | MCM with lognorm | 2649.474 |
| CORRECT | regorafenib | exp | 1882.162 |
|  |  | weibull | 1802.709 |
|  |  | lognorm | 1752.309 |
|  |  | llogis | 1736.670 |
|  |  | gompertz | 1862.029 |
|  |  | ggamma | 1752.168 |
|  |  | spline with knot=1 | 1735.570 |
|  |  | spline with knot=2 | 1737.380 |
|  |  | spline with knot=3 | 1649.855 |
|  |  | MCM with exp | 1884.167 |
|  |  | MCM with weibull | 1784.301 |
|  |  | MCM with ggamma | 1751.418 |
|  |  | MCM with llogis | 1737.644 |
|  |  | MCM with gompertz | 1848.322 |
|  |  | MCM with lognorm | 1754.085 |

MCM, mix cure model

eTable2: Results of scenario analysis

| **Strategy** | **Cost, $** | | **Effectiveness,**  **LY** | | **Effectiveness, QALY** | **ICER, $/QALY^a^** | |
| --- | --- | --- | --- | --- | --- | --- | --- |
| 10% of patients elected to receive best supportive care after progressing from second-line treatment | |  | |  | | |  |
| No pembrolizumab | 167178.9 | | 2.145885 | | 1.479577 | - | |
| First-line pembrolizumab | 211799.5 | | 6.082483 | | 4.88748 | 13093 | |
| Second-line pembrolizumab | 267753.2 | | 5.508432 | | 3.68216 | Dominated | |
| 30% of patients elected to receive best supportive care after progressing from second-line treatment | |  | |  | | |  |
| No pembrolizumab | 127147 | | 1.683376 | | 1.141256 | - | |
| First-line pembrolizumab | 164591.3 | | 4.800447 | | 3.833205 | 13909.71 | |
| Second-line pembrolizumab | 208478.5 | | 4.332786 | | 2.880541 | Dominated | |

eFigure 1 Microsimulation model depicting the treatment arms


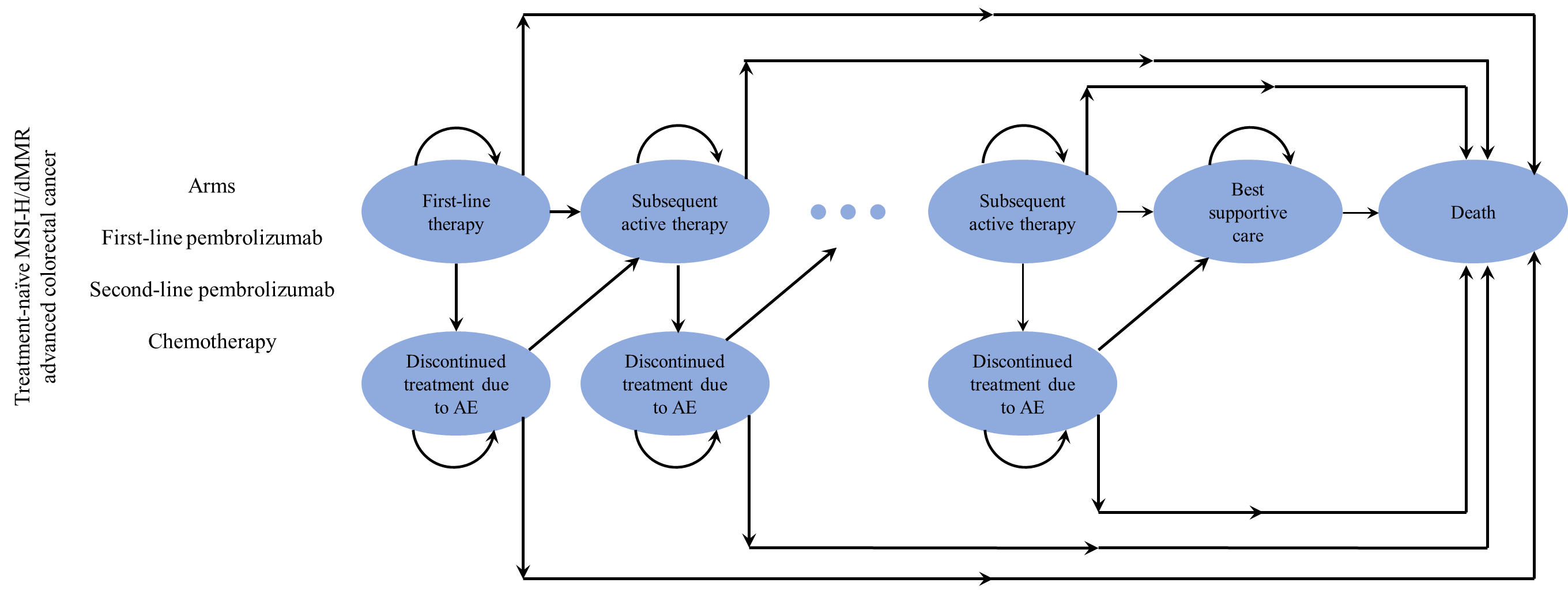


eFig 2 Parametric distributions for first-line treatment.


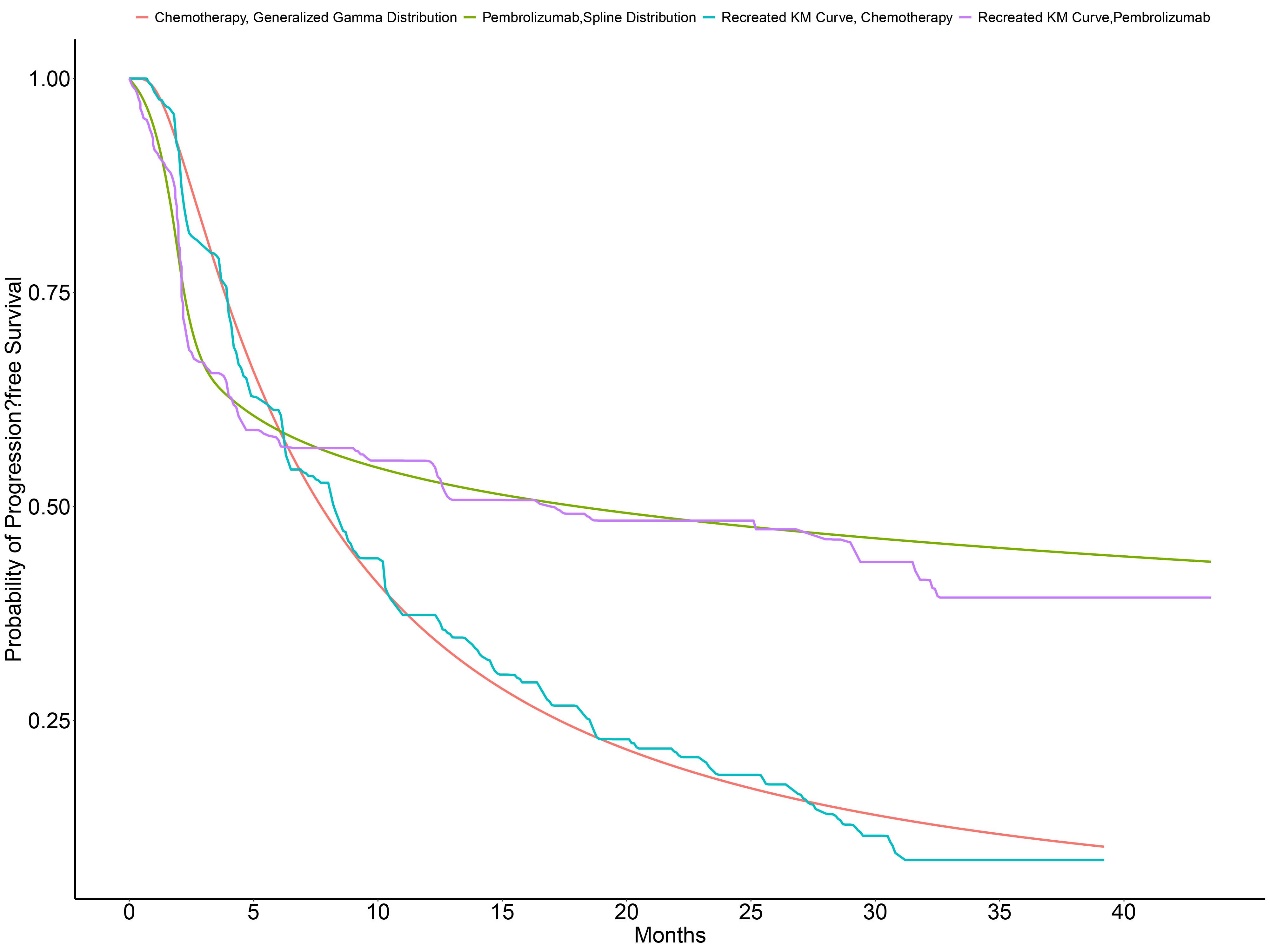


eFig 3 Parametric distributions for second-line treatment.


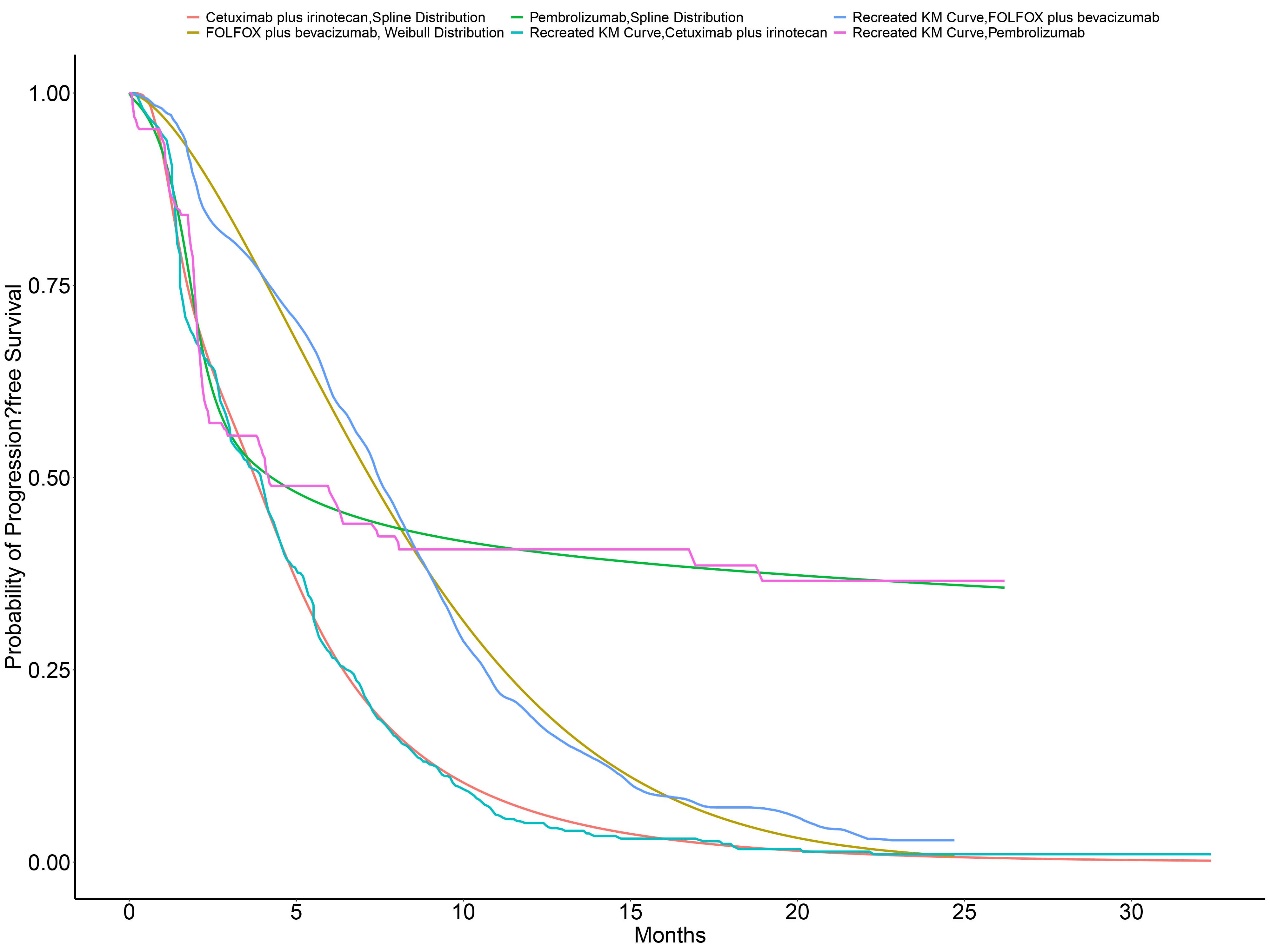


eFig 4 Parametric distributions for encorafenib plus cetuximab.


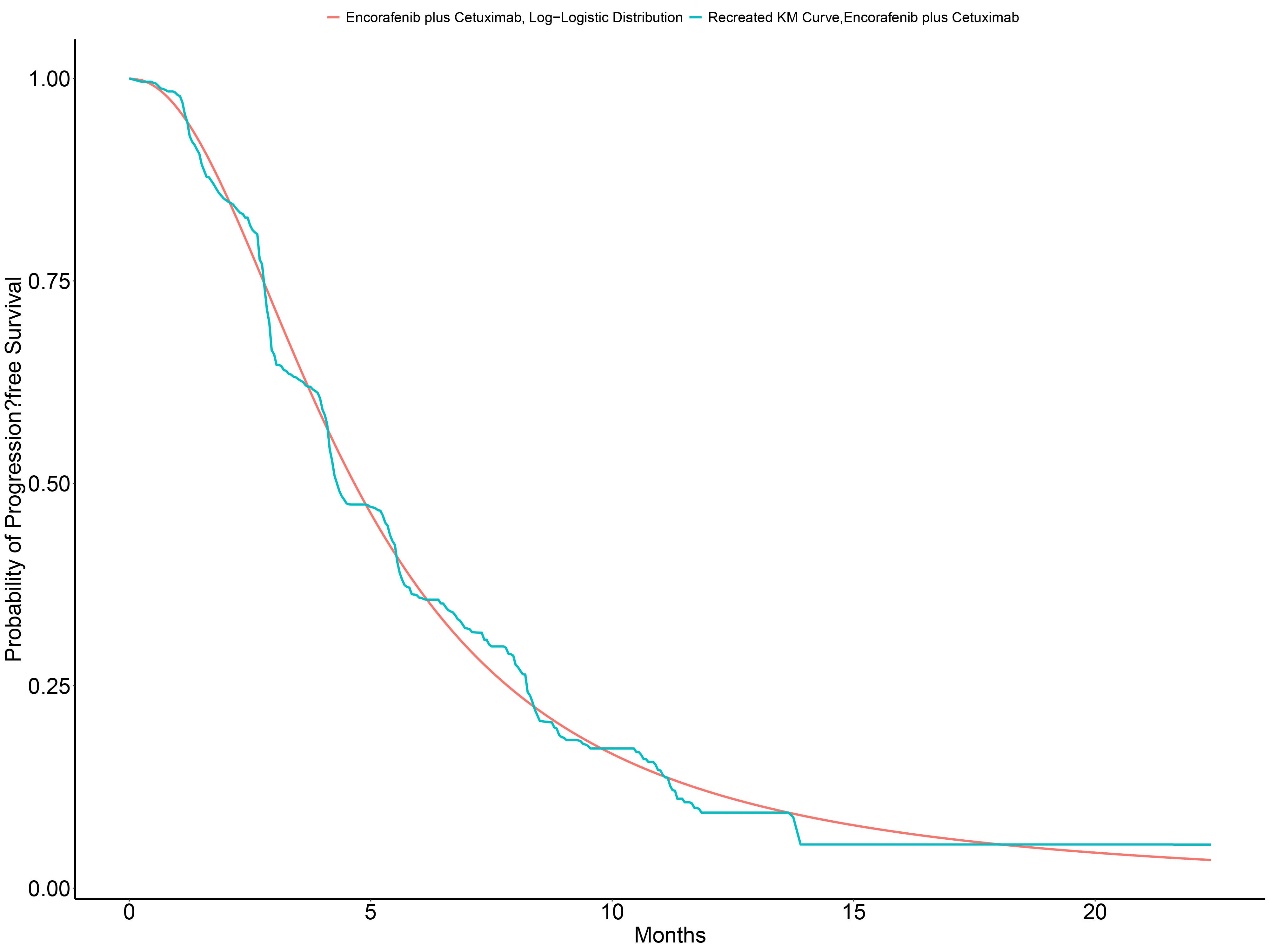


eFig 5 Parametric distributions for third-line treatment.


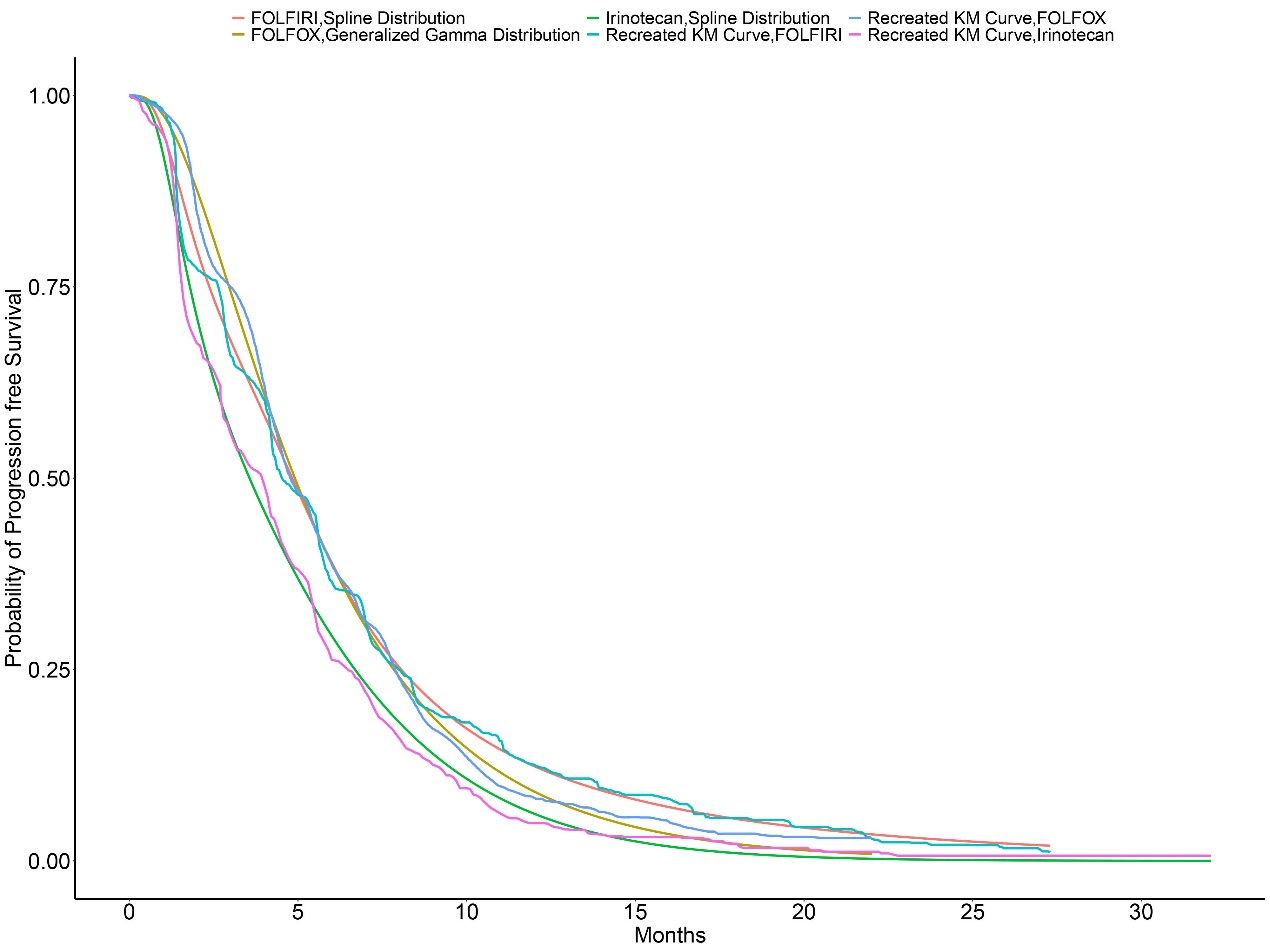


eFig 6 Parametric distributions for regorafenib.


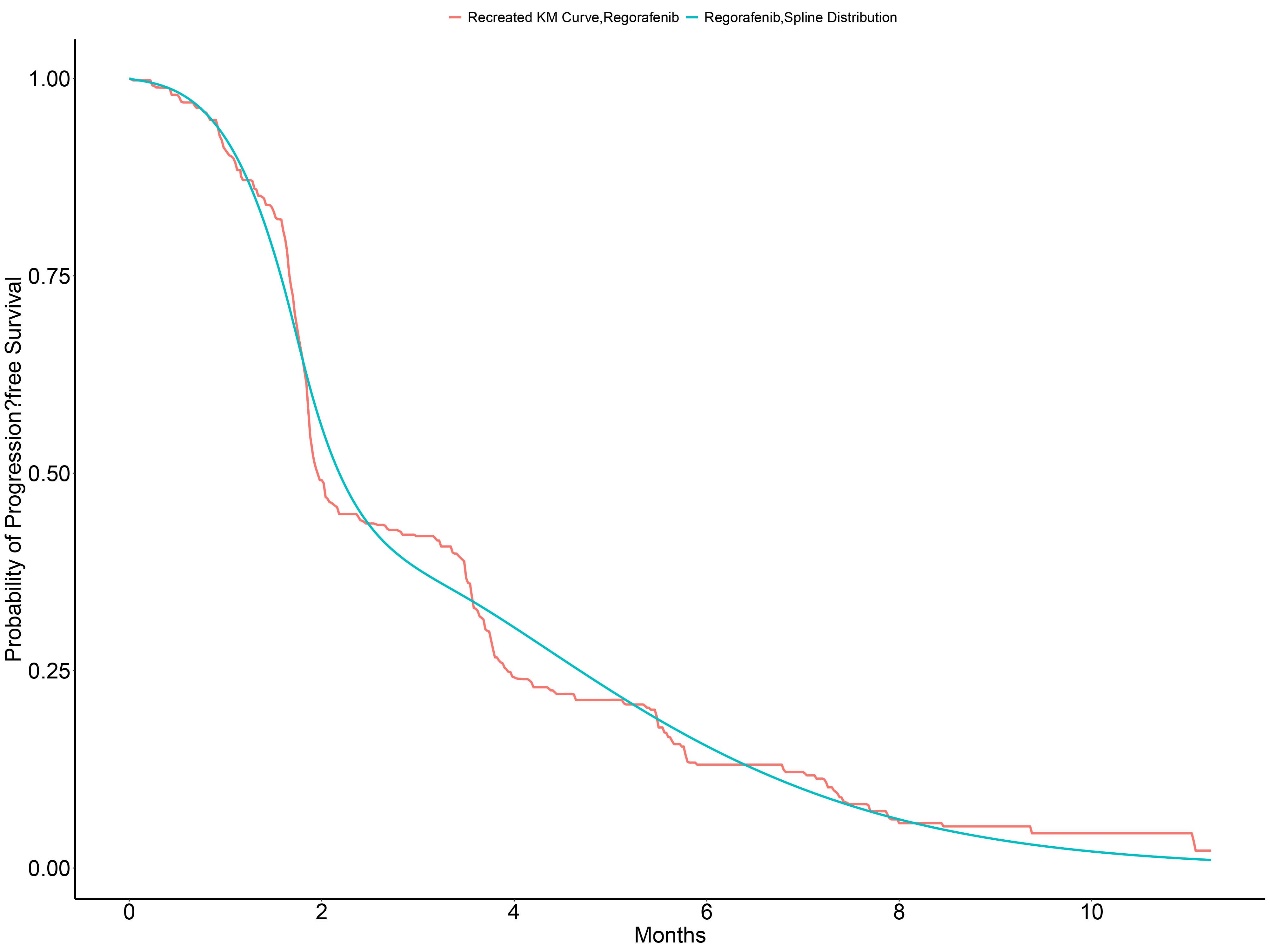

Supplement: Supplementary file 1 [file DataSheet1.docx]
